# Monitoring and Mapping Winter Wheat Spring Frost Damage with MODIS Data and Statistical Data

**DOI:** 10.3390/plants12233954

**Published:** 2023-11-24

**Authors:** Di Chen, Buchun Liu, Tianjie Lei, Xiaojuan Yang, Yuan Liu, Wei Bai, Rui Han, Huiqing Bai, Naijie Chang

**Affiliations:** 1 Institute of Environment and Sustainable Development in Agriculture, Chinese Academy of Agricultural Sciences, Beijing 100081, China; chendi01@caas.cn (D.C.); leitianjie@caas.cn (T.L.); yangxiaojuan@caas.cn (X.Y.); liuyuan@caas.cn (Y.L.); baiwei@caas.cn (W.B.); hanrui@caas.cn (R.H.); baihuiqing@caas.cn (H.B.); 2 National Engineering Laboratory of Efficient Crop Water Use and Disaster Reduction, Chinese Academy of Agricultural Sciences, Beijing 100081, China; 3 Institute of Agricultural Resources and Regional Planning, Chinese Academy of Agricultural Sciences, Beijing 100081, China

**Keywords:** frost damage, winter wheat, remote sensing, statistical data, damage monitoring

## Abstract

Spring frost is an extreme temperature event that poses a significant threat to winter wheat production and consequently jeopardizes food security. In the context of climate change, the accelerated phenology of winter wheat due to global warming advances the frost-sensitive stage, thereby escalating the risk of spring frost damage. Present techniques for monitoring and assessing frost damage heavily rely on meteorological data, controlled field experiments and crop model simulations, which cannot accurately depict the actual disaster situation for winter wheat. In this study, we propose a novel method that utilizes remote sensing index and statistical data to ascertain the spatial distribution of spring frost damage to winter wheat and evaluate the extent of damage. This method was employed to monitor and assess the spring frost damage event that occurred in Shandong province from 3 to 7 April 2018. The result shows that beginning on 3 April, the daily minimum temperature in western Shandong Province dropped significantly (decreased by 17.93 °C), accompanied by precipitation. The daily minimum temperature reached the lowest on 7 April (−1.48 °C). The growth of winter wheat began to be inhibited on 3 April 2018, and this process persisted until 13 April. Subsequently, the impact of spring frost damage on winter wheat ceased and growth gradually resumed. The affected area of winter wheat spanned 545,000 mu with an accuracy rate of 89.72%. Severely afflicted areas are mainly located in the cities of Jining, Zaozhuang, Dezhou, Heze, Liaocheng, Jinan and Tai’an in western Shandong province, and the yield reduction rates were 5.27~12.02%. Our monitoring results were consistent with the distribution of county-level winter wheat yield in 2018 in Shandong province, the daily minimum temperature distribution during spring frost and severely afflicted areas reported by the news. This method proves effective in delineating the spatial distribution of agricultural disasters and monitoring the extent of disaster damage. Furthermore, it can provide reliable information of disaster area and geospatial location for the agricultural department, thereby aiding in disaster damage assessment and post-disaster replanting.

## 1. Introduction

In recent decades, the escalating global climate change has amplified the frequency and intensity of extreme weather events, thereby exacerbating agricultural disasters [1,2,3]. Winter wheat, a pivotal crop in the Huang-Huai Plain, is instrumental in safeguarding China’s food security [4,5]. However, during the elongation stage in spring, a precipitous drop in temperature causes canopy temperatures to plummet below 0 °C or Stevenson screen air temperatures to drop below 2 °C, which inflict damage on the plants, potentially leading to their demise, a phenomenon known as spring frost damage [6]. Such damage can jeopardize the normal growth of winter wheat and result in significant reductions in both crop yield and quality [7,8]. The advancing phenology of winter wheat due to global warming and an increase in warm winter years has made spring frost a significant threat to winter wheat production. This has implications for grain production in the Huang-Huai Plain [9,10,11]. Therefore, an accurate understanding of the spatial distribution and post-disaster losses of spring frost disaster in the Huang-Huai Plain is crucial for effective disaster prevention and mitigation, and for ensuring food security. This knowledge can also assist agricultural agencies and farmers in implementing necessary measures to minimize production and economic losses.

Most studies on spring frost damage of winter wheat have mainly relied on analysis of meteorological data and crop yield data, field control experiments and crop model simulations [1,12,13,14,15,16]. However, the low spatial resolution of meteorological and crop yield data limits the accurate representation of disaster spatial distribution. Field control experiment can directly analyze the physiological and ecological mechanism of spring frost damage, but this method overlooks the compound influence of other factors and fails to reflect the actual conditions in the natural environment [7]. Recent advancements have seen the application of hyperspectral techniques and image-based computational learning techniques in field control experiments [17,18,19]. Despite providing valuable insights, these studies are confined to field trials and cannot be broadly applied to large-scale crop monitoring due to instrument limitations and complex atmospheric conditions. Several crop models, such as APSIM-Wheat [20], FROSTOL [21] and Ceres-Wheat [22], have been successfully utilized to simulate freezing damage in wheat production. These models effectively handle the interaction between spring frost and other factors, providing significant insights into wheat frost damage mechanisms, which are crucial for early warning and post-disaster evaluation [22]. However, these models fall short in fully explaining the effects of extreme weather events on crop growth. Additionally, their applicability is often limited to specific small areas, introducing considerable uncertainty into simulation results [23,24].

Remote sensing technology has become one of the most powerful tools for monitoring crop growth and evaluating the impact of natural disasters on agriculture because of its objective, large-scale and multi-temporal characteristics [9]. The normalized difference vegetation index (NDVI) is the most frequently employed remote sensing vegetation index for crop growth monitoring [25,26]. Physiological changes within the winter wheat plant following spring frost can result in significant variations in the spectral response of winter wheat [27]. These spectral reflectance variations can lead to a sharp decline in the NDVI. Based on this principle, some scholars have developed remotely sensed vegetation index (VI)-based damage quantitative indicators to evaluate the extent of spring frost damage to winter wheat at the provincial scale, and achieved good results [7,8,28]. However, these studies focused on using machine learning algorithms to predict the damage situation, or analyzing how environmental factors and crop growth conditions jointly affect the spring frost disaster, and did not provide precise affected areas and the spatial distribution of the extent of the disaster, which are the monitoring results most anticipated by the official agricultural ministry. In addition, issues such as mixed pixels introduce considerable uncertainty into large-scale monitoring using remote sensing data.

To address these challenges, a case study of winter wheat spring frost damage in Shandong province in April 2018 was conducted. The aim of this study was (1) to monitor the spatio-temporal distribution of winter wheat spring frost damage and assess the extent of frost damage rapidly by using a remote sensing index at a large scale; (2) to obtain more accurate disaster area information based on quantitative monitoring of remote sensing and self-adaptive correction of statistical data. The findings of this research have significant implications for agricultural disaster prevention and reduction and for food security, offering valuable insights for agricultural departments to deploy and promote post-disaster agricultural recovery work.

## 2. Study Area and Data

### 2.1. Study Area

**Figure 1:**
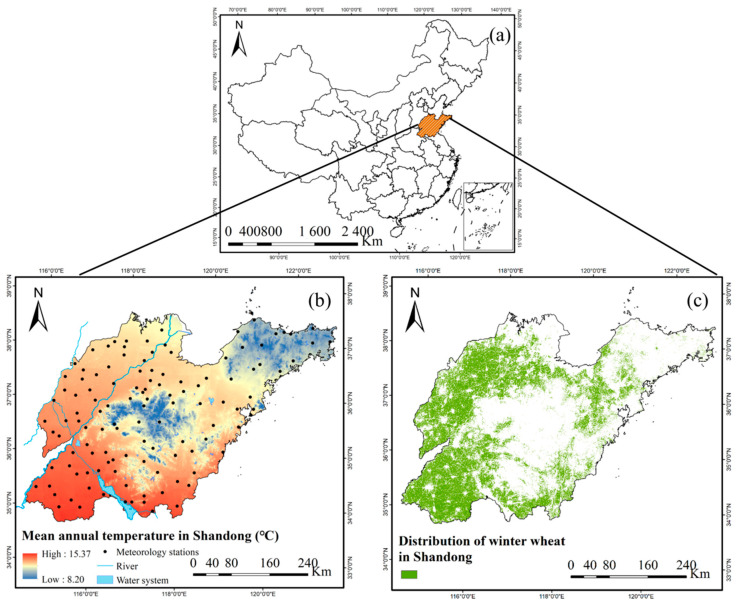
Geographical location of Shandong province, China (**a**), mean annual temperature and locations of meteorological stations (**b**), and distribution of winter wheat across the province (**c**).

Shandong province (Figure 1a) is located along the eastern coast of China, between 34° N and 38° N and between 114° E and 122° E, and is one of the main grain production areas in China. The western and northern parts of Shandong belong to the North China Plain, and the eastern, central and southern parts are mountainous and hilly. The province experiences a mean annual temperature ranging from 8 °C to 15 °C (Figure 1b). The annual frost-free period extends from 180 days along the northeast coast to 220 days in the southwest. Shandong Province has abundant light resources, with annual light hours of 2290–2890 h, and heat conditions are sufficient to double cropping annually. The mean annual precipitation is generally between 550 and 950 mm, decreasing from southeast to northwest. Approximately 60–70% of annual precipitation is concentrated in summer, which is prone to flood disaster. Drought is prone to occur in winter, spring and late autumn, which has the greatest impact on agricultural production. The cropland area accounts for 73.6% (~11.57 × 10^6^ ha) of the land area in the province, and the main crops are winter wheat (Figure 1c), corn and sweet potato.

### 2.2. Data

#### 2.2.1. MODIS Time-Series Data

Since spring frosts usually last no more than a week, the Moderate Resolution Imaging Spectroradiometer (MODIS) Version 6.1 Nadir Bidirectional Reflectance Distribution Function (BRDF)-Adjusted Reflectance (NBAR) dataset (MCD43A4), which produced daily using 16 days of Terra and Aqua MODIS data at 500 m resolution, was used for this study. The MCD43A4 contains BRDF-adjusted reflectance data for 1–7 bands and has been atmospheric corrected and converted into standard sinusoidal projections. Time series NDVI can reflect the status of the whole growth cycle of winter wheat. The winter wheat green-up period ends in March, and the frost resistance of winter wheat is greatly weakened after it enters the elongation stage in early April. In the elongation stage and heading period, the NDVI curve of winter wheat shows a continuous upward trend under normal environmental conditions. At this time, if a cold wave attacks, spring frost damage will occur, and the NDVI curve will suddenly drop, which will continue for a period of time before recovery. In the middle of May, winter wheat enters the filling stage, and the decrease in chlorophyll content leads to the decrease in NDVI, so it is not suitable to monitor the spring frost damage through the change in NDVI. Therefore, based on historical disaster event records, after excluding spring frost years in the last ten years except 2018, we selected images from 1 March to 31 May 2014 to 2021 for spring frost damage monitoring. The MODIS Reprojection Tool (MRT) was used for mosaicking and projection transformation, and finally, these original images were clipped to the scope of the study area. In this study, the original MCD43A4 data were output into Geo TIFF format and reprojected into the GCS_WGS_1984 coordinate system, and its 500 m spatial resolution remained unchanged.

#### 2.2.2. Statistical Data

The statistical survey data include the semi-monthly agricultural disaster dispatch data of the Ministry of Agriculture and Rural Affairs of Shandong Province, the relevant disaster reports of the *Shandong Rural Mass Daily* on the spring frost damage in April 2018, and relevant research literature of the meteorological bureau, agricultural bureau and agricultural technology extension station on the spring frost damage in April 2018. The statistical data of winter wheat at the county level in Shandong Province were obtained from the statistical yearbook of prefecture-level cities in Shandong Province. This study collected the data of winter wheat yield, area and yield per unit area in various counties of Shandong Province from 2014 to 2021, and used them to compare the average yield, area and yield of winter wheat in 2018 and other non-disaster-stricken years to determine the disaster-stricken counties and cities.

#### 2.2.3. Meteorological Data

The meteorological data of 123 meteorological stations in Shandong Province were collected from the Chinese Meteorological Administration and include the daily observation data of mean temperature, minimum temperature and precipitation. Meteorological data are used to identify frost-free years and to determine the start date of spring frost events.

#### 2.2.4. Winter Wheat Percentage Map

The 30 m distribution map of winter wheat planting area in Shandong province from 2016 to 2018 was derived from an open-data repository [5]. We only kept the pixels in the winter wheat planting area unchanged during the three years for subsequent analysis. Since Shandong Province is the main producing area of winter wheat, we assumed that this part of winter wheat planting area remained unchanged throughout the study period. The distribution map was obtained from Landsat 7, Landsat 8 and Sentinel-2 satellite data through a time-weighted dynamic time warping method [5]. The overall accuracy of this map is 94.49%, and the producer’s and user’s accuracy of winter wheat is 93.27% and 97.28%, respectively. The estimated winter wheat area exhibited good correlations with the agricultural statistical area data at the municipal (R^2^ = 0.95) and county levels (R^2^ = 0.75). In our study, the 30 m distribution map was resampled to 500 m (consistent with MCD43A4 data), and a 500 m resolution winter wheat percentage map was produced with the GCS_WGS_1984 coordinate system.

## 3. Methodology

### 3.1. Normalized Difference Vegetation Index Calculation

The normalized difference vegetation index (NDVI) [29] can reflect crop growth well and is one of the most widely used vegetation indices at present. The MCD43A4 was used to calculate NDVI with the following equation:

(1) $$NDVI=\frac{b_{2}-b_{1}}{b_{2}+b_{1}}$$

where $b_{1}$ is the MODIS red band and $b_{2}$ is the MODIS near-infrared band. In order to remove data noise caused by clouds and harsh atmospheric conditions, a Savitzky-Golay (SG) filter was used to smooth the vegetation index; the filter window size was 7, and the remaining parameter values were set to default.

### 3.2. Reference NDVI Curve

A time series of normalized difference vegetation index (NDVI curve) is extensively employed for qualitative and quantitative evaluation of vegetation cover and its growth status. In years without spring frost, winter wheat exhibits a normal NDVI curve, which cannot be directly obtained in a spring frost year. Consequently, the average curve of the normal NDVI curve from multiple frost-free years is designated as a candidate for the reference NDVI curve in this study. However, due to the different climatic conditions and cultivation practices across different years, the multi-year average NDVI curve does not perfectly substitute for the reference NDVI curve of a frost year. To address this, we utilized the shape model fitting (SMF) method [30] to adjust the multi-year average NDVI curve and derive the reference NDVI curve.

We defined the multi-year average NDVI curve as a shape model of the reference NDVI curve. There are still significant differences between the shape model and the SG-filtered frost-year NDVI curve in magnitude and crop growth cycle. We used Equation (2) to geometrically scale the multi-year average NDVI curve to better match the SG-filtered frost-year NDVI curve:

(2) $$h\left(x\right)=s_{y}\times \left\{g\left(s_{x}\times \left(x+t_{0}\right)\right)+bias\right\}-bias$$

where the function $g\left(x\right)$ refers to the shape model. $h\left(x\right)$ is the reference NDVI curve transformed from $g\left(x\right)$ by optimizing three scaling parameters ($s_{x}$, $s_{y}$, and $t_{0}$). $s_{x}$ and $t_{0}$ represent phenological adjustment, $s_{y}$ represents magnitude adjustment. The bias is the value fixed for each crop species. The optimal scaling parameters were obtained by minimizing the weighted root mean square error between the multi-year average NDVI curve and the SG-filtered frost-year NDVI curve. According to previous research experience, the value range of each parameter was: $S_{x}\in \left(0.9,1.1\right)$, $S_{y}\in \left(0.5,1.85\right)$ and $t_{0}\in \left(-10,10\right)$, and the bias value of winter wheat was 0.61 [7,30]. The multi-year average NDVI curve transformed by the SMF method can be considered as a reference NDVI curve, which can better reflect the growth of typical winter wheat unaffected by spring frost under similar climate and cultivation conditions as the frost year.

### 3.3. Spring Frost Damage Index

**Figure 2:**
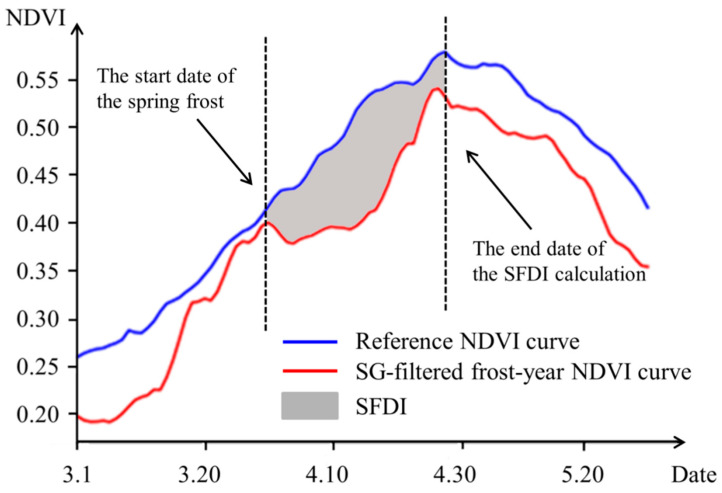
The schematic diagram of SFDI calculation. SFDI (gray shadow) is the area surrounded by the reference NDVI curve (blue) and SG-filtered frost-year NDVI curve (red) from the start of spring frost (first vertical dashed line) to the end of spring frost (second vertical dashed line). These curves were extracted from a pixel in the winter wheat planting area of northwest Shandong Province.

Spring frost has a significant impact on the growth and development of winter wheat. NDVI values drop sharply during frost, and it usually takes several weeks for the crop to return to a relatively normal level. At this time, the NDVI curve affected by spring frost. When winter wheat suffers from spring frost damage, the area enclosed by the reference NDVI curve (the blue curve in Figure 2) and the spring frost-affected NDVI curve (the red curve in Figure 2) can be regarded as an indicator of the severity of frost impact on winter wheat. Based on this, a remote sensing-based spring frost damage index (SFDI) [7] (the gray shadow in Figure 2) was used in our research. Due to the high temporal resolution of MCD43A4, SFDI has the potential for real-time computing, which allows farmers to conduct field management in a timely manner and reduce spring frost damage. The SFDI can be defined by Equation (3):

(3) $$SFDI=\sum_{i=SF_{begin}}^{i=SF_{end}}\left(NDVI_{r_{i}}-NDVI_{SF_{i}}\right)$$

where $NDVI_{r_{i}}$ is the value in the reference NDVI curve, and $NDVI_{{\mathrm{SF}}_{i}}$ is the value in the spring frost-affected NDVI curve. $SF_{begin}$ is the start date of the spring frost (marked by the first vertical dashed line in Figure 2), and $SF_{end}$ is the end date of the SFDI calculation. The end date is generally determined by the end date of the spring frost event observed in the meteorological record or one week after the spring frost event. In order to evaluate the extent of spring frost damage, the persistent effects of spring frost damage were considered in the calculation of SFDI. Because the value of the reference NDVI curve reaches its maximum when winter wheat is about to enter the grouting stage, the leaf color changes gradually after the grouting stage begins, and it is difficult to distinguish the spectral curve characteristics of frozen crops from the normal spectral curve. Therefore, we set the date when the reference NDVI curve reaches its peak as the end date of the SFDI calculation (marked by the second vertical dashed line in Figure 2).

### 3.4. Self-Adapting Statistics Correction Method

**Figure 3:**
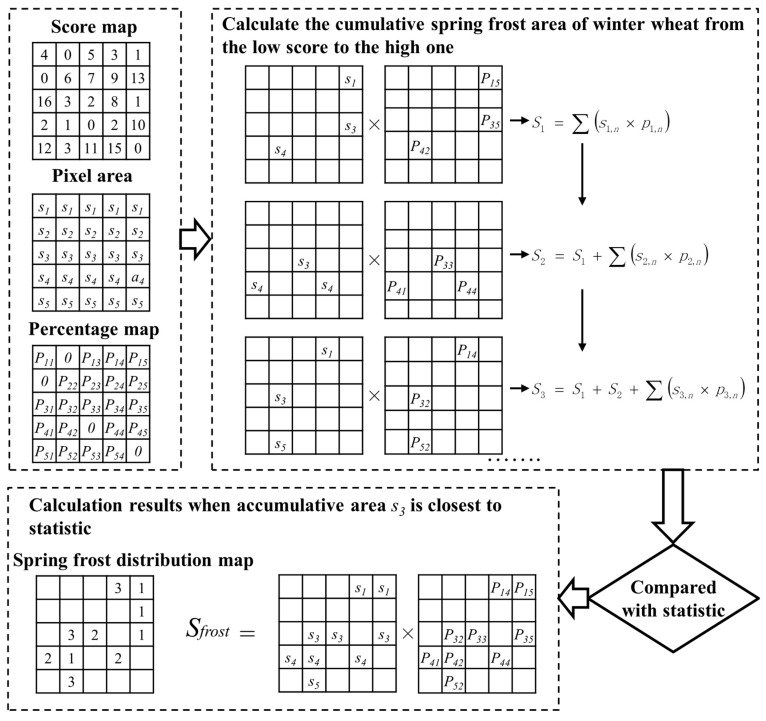
Flowchart of the self-adapting statistics correction method.

The SFDI value was calculated for each pixel, that is, the area surrounded by SG-filtered frost-year NDVI curve and reference NDVI curve of winter wheat from the beginning of spring frost (when the SG-filtered frost-year NDVI curve declined) to the end of spring frost (when the reference NDVI curve reached its peak). Because there is no uniform classification standard for the SFDI value, it is unreasonable to simply take the pixel of “SFDI > 0” as the affected area of winter wheat. In addition, the problem of mixed pixels in MODIS images may lead to inaccurate disaster identification results, so we propose a Self-Adapting Statistics Correction (SASC) Method (Figure 3) to correct the remote sensing results.

The process of the method was as follows: First, we set up a score table of SFDI. It was posited that a higher SFDI value for a pixel indicates a more severe disaster, thereby increasing the likelihood of it being a disaster area. Consequently, such pixels were assigned lower scores, ensuring their earlier inclusion in the calculation of the disaster area. The scoring rule stipulated that higher SFDI values correspond to lower scores. Based on the calculation results, all pixels with “SFDI ≥ 16” were scored as 1, all pixels with “15 ≤ SFDI < 16” were scored as 2, and so forth, until all pixels with “1 ≤ SFDI < 2” were scored as 16. The second step involved calculating the area affected by spring frost. In this step, pixels with the lowest score were selected, and their winter wheat area was calculated by multiplying the winter wheat percentage by the corresponding pixel area. Subsequently, pixels with the next lowest score were selected and added to the previous pixels. The new total area was then calculated. This process was repeated until the mapped winter wheat area closely matched the statistical area. Finally, these selected pixels represented the areas affected by spring frost in winter wheat. This process yielded a corrected winter wheat spring frost distribution map and the affected area of winter wheat.

### 3.5. Validation Method

In this study, we employed a method of combining qualitative and quantitative analyses, to validate the accuracy of remote sensing extraction results pertaining to frost damage in winter wheat. The qualitative analysis involved a comparative study of the disaster area, region, and severity against official statistics and meteorological records. For the quantitative analysis, we posited that the accuracy of the extraction results is directly proportional to the consistency between the actual disaster area and the monitored disaster area. Consequently, we defined the accuracy rate as the ratio of the actual disaster area to the monitored disaster area. This accuracy rate was computed using Equation (4):

(4) $$P=\left\{\begin{array}{c} \frac{Area_{official}}{Area_{monitoring}}\times 100\%\\ \frac{Area_{monitoring}}{Area_{official}}\times 100\% \end{array}\right.\begin{array}{c} Area_{monitoring}\geq Area_{official}\\ Area_{monitoring}<Area_{official} \end{array}$$

where P is the accuracy rate, $Area_{official}$ is the winter wheat frost-affected area based on official statistics surveyed by the Ministry of Agriculture and Rural Affairs, and $Area_{monitoring}$ is the winter wheat frost-affected area obtained in this study. The closer the P value is to 1, the higher the accuracy.

## 4. Results

### 4.1. April 2018 Spring Frost Event

According to historical disaster event records, Henan, Shanxi, Shaanxi, Shandong and Anhui experienced severe cooling weather from 3 to 7 April 2018, with a temperature drop of approximately 15 °C. According to the meteorological department report, from 5 to 7 April, the lowest temperature in each city and county was 1 °C, 0 °C, and −1 °C, respectively. The lowest temperature on the ground was −4.5 °C, and the grass surface temperature dropped to −8.5 °C. During this period, the growth of winter wheat was in the late elongation stage to booting stage. The sharp drop in temperature caused meiosis in the pollen mother cells and reduction in pollen grains of young ears. This led to a decrease in the grain number per ear of winter wheat, ultimately resulting in a reduction in yield.

### 4.2. Spatio-Temporal Distribution of Winter Wheat Spring Frost Damage

In this study, the winter wheat SG-filtered NDVI curve (the red curve in Figure 2) and reference NDVI curve (the blue curve in Figure 2) of non-disaster-stricken years were extracted from a specific pixel in a winter wheat planting area in northwest Shandong Province from 1 March to 31 May 2018. As depicted in Figure 2, the winter wheat SG-filtered NDVI curve began to exhibit a slight downward trend on the 33rd day (3 April 2018), indicating inhibited growth of winter wheat. After the 43rd day (13 April 2018), the SG-filtered NDVI curve of winter wheat began to rise, marking the end of the impact of spring frost damage on winter wheat and the resumption of its growth. Through the calculation of the SFDI and Self-adapting Statistics Correction Method, it was determined that the area of winter wheat affected by spring frost was 545,000 mu.

**Figure 4:**
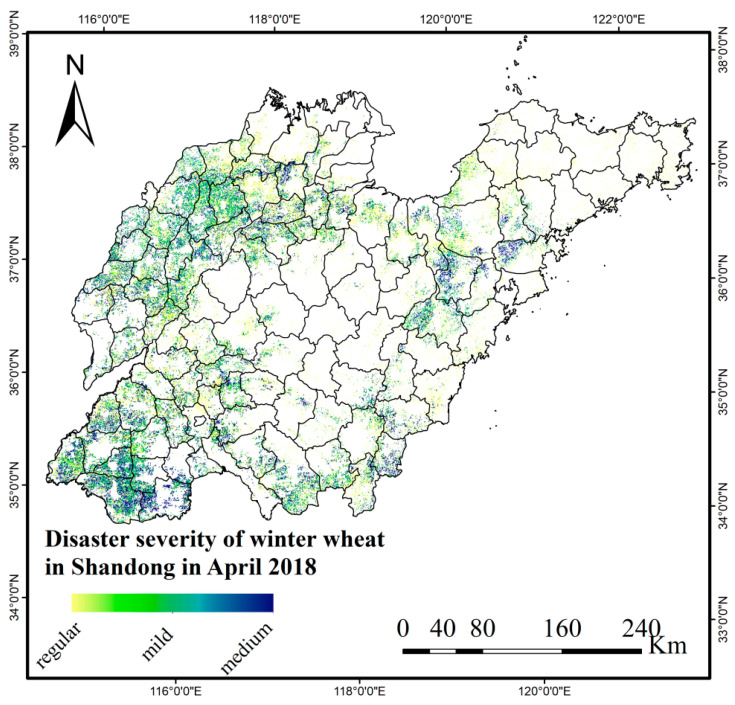
The extent and severity of winter wheat spring frost damage in April 2018 in Shandong Province.

The crop affected area refers to the sown area where crop production is reduced by more than 10% compared to the normal year due to the disaster [31]. The crop disaster area refers to the sown area where crop production is reduced by ≥30% compared with normal years, due to disasters [31]. The survey of spring frost damage of winter wheat in Shandong Province in April 2018 by the Ministry of Agriculture and Rural Affairs only reported the affected area. Therefore, it was inferred that the loss of winter wheat production during the spring frost process was between 10% and 30%. According to the classification standard of wheat low temperature freezing damage in the *Emergency Plan for Major Agricultural Natural Disasters* formulated by the Ministry of Agriculture and Rural Affairs [32], a wheat yield reduction of less than 10% is considered mild freezing damage, while a wheat yield reduction of between 11% and 30% is considered medium freezing damage. The disaster severity of winter wheat freezing damage during spring frost in Shandong Province in April 2018 is depicted in Figure 4.

As can be seen from Figure 4, the damage severity of winter wheat in the west of Shandong Province was obviously higher than that in other areas, and the freezing damage was mainly distributed in the cities of Dezhou, Heze, Liaocheng, Jinan, Tai ’an, Jining, Zaozhuang, Linyi, Binzhou and Zibo. More serious counties included: Qihe county, Ling county and Xiajin county (Dezhou city); Linqing city, Chiping county (Liaocheng city); Shan county, Cao county, Dingtao district, Juancheng county and Dongming county (Heze city); Wenshang county, Qufu city and Zoucheng city (Jining city); Shanghe county (Jinan city); Ningyang county, Dongping county (Tai ’an city); Zaozhuang city; Linshu county (Linyi city); Zouping city (Binzhou city); GaoQing county (Zibo city).

### 4.3. Validation

**Figure 5:**
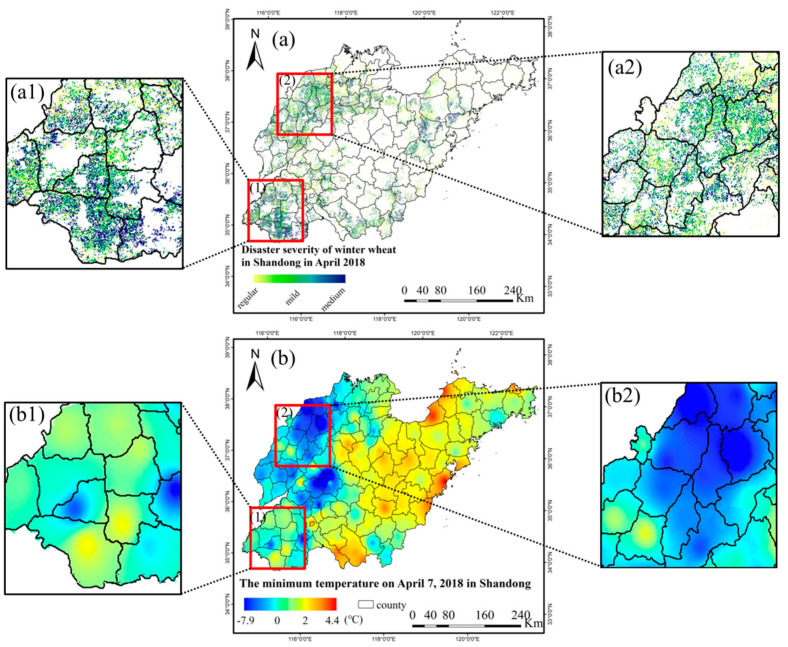
Comparison of winter wheat damage extent (**a**) and distribution of daily minimum temperature (**b**) in Shandong Province during the spring frost. The extent of damage (**a1**) and the daily minimum temperature (**b1**) in the southwest Shandong, and the extent of damage (**a2**) and the daily minimum temperature (**b2**) in the northwest Shandong are highlighted (range in red boxes).

In order to validate the accuracy of remote sensing extraction results of winter wheat spring frost damage, this study compared the disaster area, disaster region and disaster severity with official statistics and meteorological records. Official statistics show that the winter wheat frost-affected area of Shandong Province, as surveyed by the Ministry of Agriculture and Rural Affairs from 1 to 15 April 2018, was 489,000 mu. In this study, the affected area of winter wheat was 545,000 mu, and the accuracy rate was 89.72%. Meteorological records show that on 7 April 2018, the temperature dropped to the lowest point. The distribution of minimum temperature in Shandong Province on 7 April 2018 is shown in Figure 5b. The daily minimum temperature in the cities of Dezhou, Liaocheng, Jinan, Tai ’an, Jining and Heze in western Shandong was significantly lower than that of other regions. As can be seen from Figure 5(b1,b2), the temperature in Leling city, Ningjin county, Ling county, Pingyuan county, Yucheng city, Qihe county, Linyi county (Dezhou city), Dingtao county, Mudan district, Cao county (Heze city), Shanghe county (Jinan city) dropped significantly, which aligned with the distribution of winter wheat frost-damage severity obtained by remote sensing images (Figure 5(a1,a2)).

**Figure 6:**
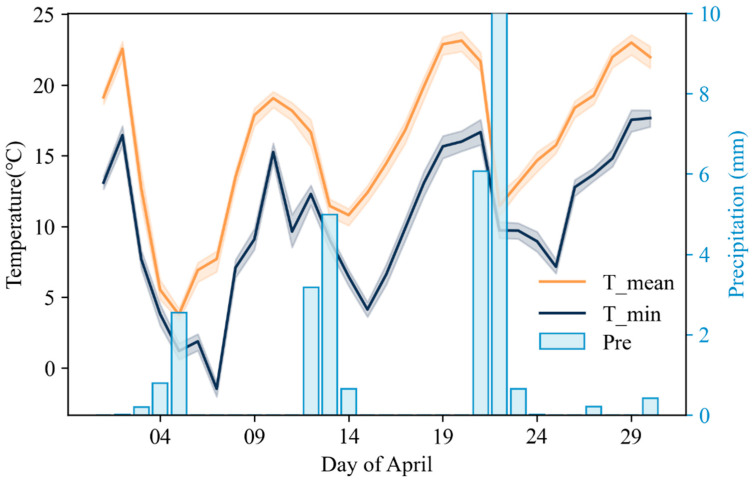
Changes in minimum temperature, daily mean temperature and daily precipitation in western Shandong Province in April 2018.

The daily minimum temperature in western Shandong Province on 7 April 2018 was significantly lower than that in other regions, and the remote sensing monitoring results showed that winter wheat in this part of the province suffered more severe freezing damage. Therefore, we analyzed the variation trends in daily minimum temperature, daily mean temperature and daily precipitation in the western part of Shandong Province (including the cities of Heze, Jining, Liaocheng, Dezhou, Jinan and Tai ’an) in April 2018, as shown in Figure 6. From 3 to 7 April, the daily minimum temperature and daily mean temperature in western Shandong Province dropped significantly, accompanied by precipitation. The daily minimum temperature reached the lowest on 7 April, which was −1.48 °C. Compared with the temperature before 2 April, it dropped by 17.93 °C The daily mean temperature reached its lowest level (3.76 °C) on 5 April, dropping by 18.8 °C compared with the temperature before 2 April. The precipitation was mainly concentrated on 5 April, with a daily precipitation of 2.55 mm. Comparing the daily minimum temperature and daily mean temperature from 3 to 7 April with the winter wheat SG-filtered NDVI curve extracted by remote sensing from March to May 2018 (the red curve in Figure 2), it can be seen that the SG-filtered NDVI curve of winter wheat began to show a slight downward trend on the 33rd day (3 April), and the growth of winter wheat was inhibited. This trend corresponded to the sharp decline in temperature on 3 April.

**Figure 7:**
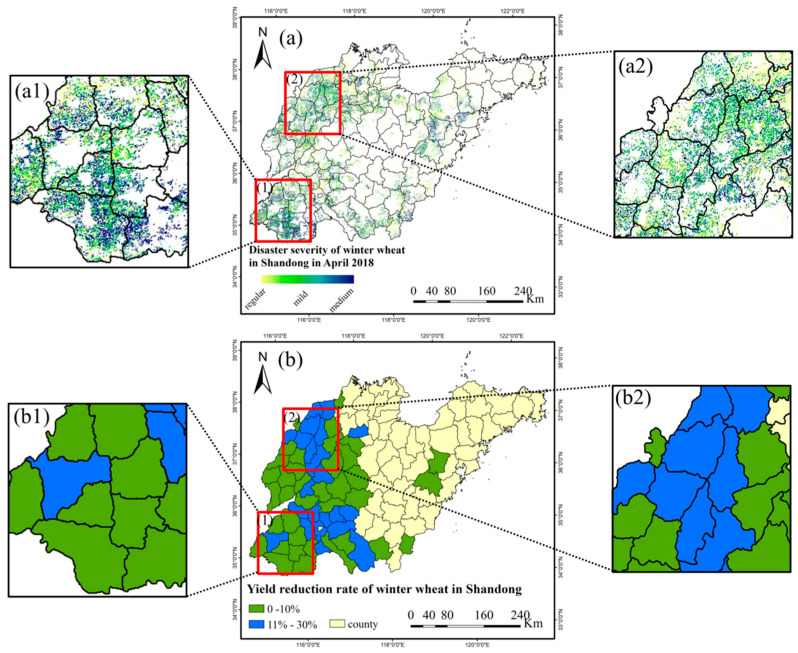
Comparison of winter wheat damage extent (**a**) and the yield reduction rate of winter wheat at the county level (**b**) in Shandong Province in 2018. The extent of damage (**a1**) and the yield reduction rate of winter wheat at the county level (**b1**) in the southwest Shandong, and the extent of damage (**a2**) and the yield reduction rate of winter wheat at the county level (**b2**) in the northwest Shandong are highlighted (range in red boxes).

At the same time, we collected county-level statistical data of winter wheat in Shandong Province from 2014 to 2020. We compared the average area, production and yield per unit area in non-disaster-stricken years with that in 2018, and calculated the yield reduction rate, as shown in Figure 7b. In 2018, the yield reduction in winter wheat in Shandong Province mainly occurred in the western region, such as Jining (reduced by 12.02%), Zaozhuang (reduced by 11.71%), Dezhou (reduced by 11.33%), Heze (reduced by 7.12%), Liaocheng (reduced by 7.11%), Jinan (reduced by 5.61%), Tai ’an (reduced by 5.27%), and some areas of Linyi and Zibo. Among these, the yield reduction rate of Leling city, Ningjin county, Ling county, Wucheng county, Pingyuan county, Yucheng city, Qihe county, Linyi county (Dezhou city), Changqing district, Pingyin county (Jinan city), Liangshan county, Jiaxiang county, Wenshang county, Rencheng district, Qufu city, Sishui county, Yanzhou district, Yutai county, Zoucheng city (Jining city), Gaoqing county (Zibo city), Heze city, and Zaozhuang city ranged between 11% and 30%. By comparing Figure 7a,b, it can be observed that the distribution characteristics of the yield reduction rate calculated by statistical data align closely with the disaster severity identified by remote sensing. In particular, as shown in Figure 7(a1,b1,a2,b2), the extent of spring frost disaster in Ling county, Pingyuan county, Yucheng city, Linyi county (Dezhou city), Shanghe county (Jinan city), Mudan district and Cao county (Heze city) have a high consistency with the distribution of yield reduction rate.

Furthermore, in order to evaluate the reliability of the results, on the basis of comparison with statistical data and meteorological data, we also searched the relevant news media reports on the spring frost disaster in April 2018. We found reports from Cao county (https://mp.weixin.qq.com/s/nDigzvn3Oy0lMSM59_AZ_w, accessed on 1 August 2023) and Liaocheng city (http://liaocheng.dzwww.com, accessed on 1 August 2023) in Shandong province that described how winter wheat was affected by strong cooling from elongation stage to booting stage, and the spike grain decreased, resulting in a significant reduction in yield. These reports were consistent with our monitoring results, thereby indirectly validating the reliability of our findings.

**Figure 8:**
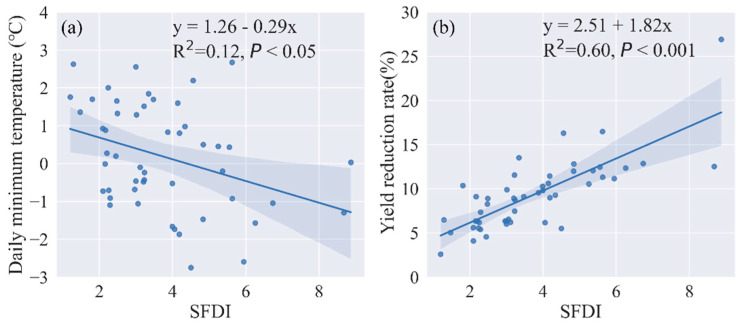
Scatterplot between the SFDI and the daily minimum temperature (**a**), the SFDI and the yield reduction rate (**b**).

In addition to the spatio-temporal pattern comparison, we also conducted a regression analysis of the SFDI with the daily minimum temperature on 7 April and the yield reduction rate, respectively, at the county level. This was done to validate the effectiveness of SFDI in reflecting crop reduction caused by spring frost. The scatterplot of SFDI and daily minimum temperature, as well as SFDI and yield reduction rate, is depicted in Figure 8a,b. There was a significant negative correlation between SFDI and daily minimum temperature (*p* < 0.05), and a significant positive correlation between SFDI and yield reduction (*p* < 0.001). The R^2^ value was 0.12 for the correlation between SFDI and the daily minimum temperature, and 0.60 for the correlation between SFDI and the yield reduction rate. These results indicate a significant association between the daily minimum temperature and freezing damage of winter wheat, and that SFDI could reflect the yield loss caused by spring frost.

## 5. Discussion

Previous studies have analyzed the physiological mechanism behind the decline in VI due to frost, finding that frost damage to vegetation can be identified by the reduced VI value [33,34]. NDVI is globally recognized as a reliable index for monitoring vegetation growth and evaluating vegetation productivity [9]. Following exposure to spring frost, the internal tissue structure of winter wheat is damaged, leading to a significant decrease in leaf chlorophyll content. This results in an increase in the reflectance of winter wheat in the red band and a decrease in the green band. At this time, the position of the “red edge” was significantly shifted to the blue band, that is, the “blue shift” had occurred. Simultaneously, as the temperature dropped below 0 °C, ice crystals formed between cells, and the reflectance of the near infrared band decreased due to changes in cell structure [7]. These phenomena collectively result in a sharp decline in NDVI. Of course, other vegetation indices derived from remote sensing data, such as EVI and NDPI, have also been selected in some studies and achieved good monitoring effects [7,11,26]. Their principles are similar to those of NDVI, hence they are not detailed further in this paper.

In most studies on monitoring and evaluating frost damage of crops by using remote sensing VI, the difference between the VI curve of a frozen year and normal year or the VI curve changes before and after freezing injury were usually compared [11,24,35,36]. However, these methods merely compare differences without considering errors caused by climate and phenological differences between years, or the continuous impact of spring frost. This introduces considerable uncertainty into the monitoring and evaluation outcomes. To address these issues, SFDI considers both the entire evolution of a spring frost event (including the immediate reduction in NDVI when frost occurs) and the ongoing effects of frost on crops (the response process of winter wheat to spring frost). SFDI calculates the cumulative NDVI reduction between the reference NDVI curve and the frost-affected NDVI curve, which both reflects the ongoing impact of spring frost on the winter wheat and effectively avoids possible calculation errors on an individual date. In the construction of the SFDI, the average curve of the normal NDVI curve of multiple frost-free years was defined as a candidate for the reference NDVI curve. The SMF method was used to obtain the reference NDVI curve. The application of three optimized scaling parameters ($s_{x}$, $s_{y}$, and $t_{0}$) in the SMF method can eliminate the influence of climatic conditions, phenology and farmland management measures between different years, and make the reference NDVI curve more universal [30]. It is worth noting that the application of the SFDI is very flexible; it is not only applicable at the provincial scale, but it can also be extended to the national and global scale. It can be easily extended to other natural disasters and other crop types [7]. In contrast to field experiments and crop models, remote sensing-based methods offer several distinct advantages. They obviate the need for intricate parameter calibration and enable large-scale, real-time monitoring of extreme climatic disasters. Furthermore, they facilitate the assessment of the disaster’s impact on crops in their natural environments. These methods can provide timely data support, which is crucial for post-disaster recovery and replanting efforts, as well as for enhancing field management measures. This underscores the potential of remote sensing as a valuable tool in disaster management and agricultural planning. Since we lacked proper field survey data on actual spring frost damage to winter wheat that had occurred in the past, and there was also a lack of uniform standards for measuring SFDI, the results of remote sensing monitoring can only reflect the relative extent of spring frost damage and cannot be used to calculate the real area of spring frost damage directly. To solve this problem, we proposed the SASC method. The basic principle of this method is to use official statistics to correct remote sensing monitoring results. The statistics of the Ministry of Agriculture and Rural Affairs were derived from agricultural disaster information officers who work in the field all year round and have rich experience in judging agricultural disasters. We considered such official statistics to be of relatively high confidence and can be used to aid our calculations. The pixel with the highest SFDI value is considered to be the pixel with the highest risk of disaster and can be counted first. This process continued until the mapped winter wheat area matched the statistical area as closely as possible. This can avoid the misjudgment of the extent of disaster caused by the inconsistency of SFDI measurement. It not only ensures the accuracy of spatio-temporal information of spring frost damage, but also ensures the accuracy of area information of spring frost damage.

In this study, we utilized daily MCD43A4 data with a spatial resolution of 500 m. While the computation of the SFDI is not contingent on spatial resolution [7], it is important to note that MODIS data may not be suitable for smaller planting areas (less than 25 to 100 hectares) due to the issue of mixed pixels. For such areas, higher-resolution data, such as those from Sentinel-2 and Landsat, would be more appropriate. However, given that spring frost typically lasts no more than a week, these high spatial resolution remote sensing images do not offer a sufficiently high temporal resolution to monitor the occurrence and progression of spring frost in a timely manner. To mitigate the effect of mixed pixels, we calculated the percentage of winter wheat distribution in each 500-m pixel when resampling the 30-m winter wheat distribution map to 500 m, and applied the winter wheat percentage map to the SASC method (refer to Section 3.4) to more accurately determine the affected area of winter wheat.

However, this study still has some limitations. Different crops have varying growth cycles, hence the SFDI can only be used to assess the impact of spring frost on a single crop and is not suitable for evaluating the impact on multiple crops simultaneously. Besides spring frost, other agricultural disaster events may also reduce the NDVI value [37,38], and the SFDI reflects this cumulative impact. Therefore, without prior knowledge or assumptions, it is challenging to distinguish damage caused solely by a spring frost event. The occurrence and start dates of spring frost events need to be known in advance from weather records or forecasts, and crop damage statistics need to be obtained to aid in calculations. This suggests that this method may be more suitable for describing the spatial distribution of agricultural disasters and monitoring the extent of disaster damage, and providing accurate spatial information support for disaster damage assessment and post-disaster replanting work, rather than forecasting disasters. There was a lack of field survey data during spring frost events to directly verify the results, so verification could only be done indirectly through meteorological data, statistics, and news reports. At present, mobile communication equipment is very intelligent, and we propose conducting pilot projects in several major grain provinces to fully mobilize the enthusiasm of farmers. Provincial agricultural departments should establish a crowdsourced database of agricultural disaster samples. The government departments could then organize the development of mobile phone apps, and propose that farmers upload local disaster photos and geographical locations in real time when crops are affected, and agricultural disaster information officers could select suitable photos and collect them into the disaster sample database, offering corresponding rewards or subsidies to the photographers. This not only provides first-hand information for future agricultural disaster research, but also enriches the historical disaster dataset. It is of great significance for the construction of disaster prevention and reduction in agriculture and rural areas and the realization of “smart agriculture”.

## 6. Conclusions

In this study, we introduced a methodology to determine the spatial distribution of winter wheat spring frost damage and assess the damage extent using a remote sensing index and statistical data. This method was easy to implement, and was more suitable for describing the spatial distribution of agricultural disaster and monitoring the extent of disaster damage. Moreover, it can also provide reliable disaster area and geospatial information for the agricultural department, and contribute to the development of disaster damage assessment and post-disaster replanting. We applied this method to analyze the spring frost damage in Shandong Province from 3 to 7 April 2018, and evaluated the effectiveness of this method in monitoring the area, spatial distribution and extent of spring frost damage in winter wheat through meteorological data, statistical data and news media information. The results indicated that the growth of winter wheat began to be inhibited on 3 April 2018, and this process continued until 13 April, when the effect of spring frost damage on winter wheat ended and winter wheat gradually resumed growth. The affected area of winter wheat was 545,000 mu, with an accuracy rate of 89.72%. Severely afflicted areas were mainly concentrated in the cities of Dezhou, Liaocheng, Jinan, Tai’an, Jining and Heze in western Shandong province. Our monitoring results were consistent with the distribution of county-level winter wheat yield in 2018 in Shandong province, the daily minimum temperature distribution during spring frost and severely afflicted areas reported by the news.

## Data Availability

Data are contained within the article.

## References

[1] Xiao L., Liu L., Asseng S., Xia Y., Tang L., Liu B., Cao W., Zhu Y. (2018). Estimating spring frost and its impact on yield across winter wheat in China. Agric. For. Meteorol..

[2] Li Z., Zhang Z., Zhang J., Luo Y., Zhang L. (2021). A new framework to quantify maize production risk from chilling injury in Northeast China. Clim. Risk Manag..

[3] Liu K., Harrison M.T., Yan H., Liu D., Meinke H., Hoogenboom G., Wang B., Peng B., Guan K., Jaegermeyr J. (2023). Silver lining to a climate crisis in multiple prospects for alleviating crop waterlogging under future climates. Nat. Commun..

[4] Guo C., Tang Y., Lu J., Zhu Y., Cao W., Cheng T., Zhang L., Tian Y. (2019). Predicting wheat productivity: Integrating time series of vegetation indices into crop modeling via sequential assimilation. Agric. For. Meteorol..

[5] Dong J., Fu Y., Wang J., Tian H., Fu S., Niu Z., Han W., Zheng Y., Huang J., Yuan W. (2020). Early-season mapping of winter wheat in China based on Landsat and Sentinel images. Earth Syst. Sci. Data.

[6] Frederiks T., Christopher J., Sutherland M., Borrell A. (2015). Post-head-emergence frost in wheat and barley: Defining the problem, assessing the damage, and identifying resistance. J. Exp. Bot..

[7] Wang S., Chen J., Rao Y., Liu L., Wang W., Dong Q. (2020). Response of winter wheat to spring frost from a remote sensing perspective: Damage estimation and influential factors. ISPRS J. Photogramm. Remote Sens..

[8] Zhao L., Li Q., Zhang Y., Wang H., Du X. (2020). Normalized NDVI valley area index (NNVAI)-based framework for quantitative and timely monitoring of winter wheat frost damage on the Huang-Huai-Hai Plain, China. Agric. Ecosyst. Environ..

[9] Bascietto M., Bajocco S., Mazzenga F., Matteucci G. (2018). Assessing spring frost effects on beech forests in Central Apennines from remotely-sensed data. Agric. For. Meteorol..

[10] Zheng D., Yang X., Mínguez M.I., Mu C., He Q., Wu X. (2018). Effect of freezing temperature and duration on winter survival and grain yield of winter wheat. Agric. For. Meteorol..

[11] Cogato A., Meggio F., Collins C., Marinello F. (2020). Medium-Resolution Multispectral Data from Sentinel-2 to Assess the Damage and the Recovery Time of Late Frost on Vineyards. Remote Sens..

[12] Zhang X., Yu W., Wang C. (2012). Risk evaluation for spring frost disaster of winter wheat in Yellow river-Huai river regions based on crop model. Plateau Meteorol..

[13] Li X., Pu H., Liu F., Zhou Q., Cai J., Dai T., Cao W., Jiang D. (2015). Winter wheat photosynthesis and grain yield responses to spring freeze. Agron. J..

[14] Yue Y., Zhou Y., Wang J., Ye X. (2016). Assessing wheat frost risk with the support of GIS: An approach coupling a growing season meteorological index and a hybrid fuzzy neural network model. Sustainability.

[15] Guo J., Bai X., Shi W., Li R., Hao X., Wang H., Gao Z., Guo J., Lin W. (2021). Risk assessment of freezing injury during overwintering of wheat in the northern boundary of the Winter Wheat Region in China. PeerJ.

[16] Chen R., Wang J., Li Y., Song Y., Huang M., Feng P., Qu Z., Liu L. (2023). Quantifying the impact of frost damage during flowering on apple yield in Shaanxi province, China. Eur. J. Agron..

[17] Wu Y., Ma Y., Hu X., Ma J., Zhao H., Ren D. (2021). Narrow-waveband spectral indices for prediction of yield loss in frost-damaged winter wheat during stem elongation. Eur. J. Agron..

[18] Shammi S., Sohel F., Diepeveen D., Zander S., Jones M. (2023). Machine learning-based detection of frost events in wheat plants from infrared thermography. Eur. J. Agron..

[19] Mao Y., Li H., Wang Y., Fan K., Shen J., Zhang J., Han X., Song Y., Bi C., Sun L. (2023). Low temperature response index for monitoring freezing injury of tea plant. Front. Plant Sci..

[20] Zheng B., Chapman S., Christopher J., Frederiks T., Chenu K. (2015). Frost Trends and their Estimated Impact on Yield in the Australian Wheatbelt. Procedia Environ. Sci..

[21] Bergjord Olsen A., Persson T., de Wit A., Nkurunziza L., Sindhøj E., Eckersten H. (2018). Estimating winter survival of winter wheat by simulations of plant frost tolerance. J. Agron. Crop Sci..

[22] Liu J., Yao W., Jiang M. (2021). Risk assessment of possible impacts of climate change and irrigation on wheat yield and quality with a modified CERES-Wheat model. J. Water Clim. Chang..

[23] Barlow K., Christy B., O’Leary G., Riffkin P., Nuttall J. (2015). Simulating the impact of extreme heat and frost events on wheat crop production: A review. Field Crops Res..

[24] Wang Z., Wang H., Feng M., Qin M., Zhang X., Xie Y., Wang C., Yang W., Xiao L., Zhang M. (2021). Study on the monitoring and classification of winter wheat freezing injury in spring based on 3S technology. J. Agric. Sci..

[25] Deng G., Zhang H., Yang L., Zhao J., Guo X., Hong Y., Wu R., Guo D. (2020). Estimating Frost during Growing Season and Its Impact on the Velocity of Vegetation Greenup and Withering in Northeast China. Remote Sens..

[26] Gabbrielli M., Corti M., Perfetto M., Fassa V., Bechini L. (2022). Satellite-Based Frost Damage Detection in Support of Winter Cover Crops Management: A Case Study on White Mustard. Agronomy.

[27] Lacoste C., Nansen C., Thompson S., Moir-Barnetson L., Mian A., McNee M., Flower K. (2015). Increased susceptibility to aphids of flowering wheat plants exposed to low temperatures. Environ. Entomol..

[28] Wang H., Huo Z., Zhou G., Wu L., Feng H. (2016). Monitoring and forecasting winter wheat freeze injury and yield from multi-temporal remotely sensed data. Intell. Autom. Soft Comput..

[29] Rouse J.W., Haas R., Schell J., Deering D. (1974). Monitoring Vegetation Systems in the Great Plains with ERTS. Proceedings of the Third Earth Resources Technology Satellite-1 Symposium.

[30] Sakamoto T. (2018). Refined shape model fitting methods for detecting various types of phenological information on major U.S. crops. ISPRS J. Photogramm. Remote Sens..

[31] Liu B., Liu Y., Zheng F., Zhu Y., Guo A., Chen D., Yang X., Mei X. (2022). Assessment regional grain yield loss based on re-examination of disaster-yield model in three northeastern provinces. Chin. J. Agrometeorol..

[32] The Ministry of Agriculture (2005). Emergency Plan for Major Agricultural Natural Disasters.

[33] Wei C., Huang J., Wang X., Blackburn G., Zhang Y., Wang S., Mansaray L. (2017). Hyperspectral characterization of freezing injury and its biochemical impacts in oilseed rape leaves. Remote Sens. Environ..

[34] Allevato E., Saulino L., Cesarano G., Chirico G.B., D’Urso G., Falanga Bolognesi S., Rita A., Rossi S., Saracino A., Bonanomi G. (2019). Canopy damage by spring frost in European beech along the Apennines: Effect of latitude, altitude and aspect. Remote Sens. Environ..

[35] Kim Y., Kimball J., Didan K., Henebry G. (2014). Response of vegetation growth and productivity to spring climate indicators in the conterminous United States derived from satellite remote sensing data fusion. Agric. For. Meteorol..

[36] Nolè A., Rita A., Ferrara A.M.S., Borghetti M. (2018). Effects of a large-scale late spring frost on a beech (*Fagus sylvatica* L.) dominated Mediterranean mountain forest derived from the spatio-temporal variations of NDVI. Ann. For. Sci..

[37] Huang J., Zhuo W., Li Y., Huang R., Sedano F., Su W., Dong J., Tian L., Huang Y., Zhu D. (2020). Comparison of three remotely sensed drought indices for assessing the impact of drought on winter wheat yield. Int. J. Digit. Earth.

[38] Zhou Y., Xiao X., Zhang G., Wagle P., Bajgain R., Dong J., Jin C., Basara J., Anderson M., Hain C. (2017). Quantifying agricultural drought in tallgrass prairie region in the U.S. Southern Great Plains through analysis of a water-related vegetation index from MODIS images. Agric. For. Meteorol..

